# What is the association between MRI and conventional radiography in measuring femoral head migration?

**DOI:** 10.1080/17453674.2020.1864124

**Published:** 2021-01-04

**Authors:** Hans-Christen Husum, Michel Bach Hellfritzsch, Mads Henriksen, Kirsten Skjaerbaek Duch, Martin Gottliebsen, Ole Rahbek

**Affiliations:** a Interdisciplinary Orthopaedics, Aalborg University Hospital, Aalborg;; b Department of Radiology, Aarhus University Hospital, Aarhus;; c Department of Orthopedics, Aarhus University Hospital, Aarhus;; d Danish Pediatric Orthopaedic Research;; e Unit of Epidemiology and Biostatistics, Aalborg University Hospital, Aalborg, Denmark

## Abstract

Background and purpose — Pelvic radiographs are traditionally used for assessing femoral head migration in residual acetabular dysplasia (RAD). Knowledge of the heightened importance of cartilaginous structures in this condition has led to increased use of MRI in assessing both osseous and cartilaginous structures of the pediatric hip. Therefore, we assessed the relationship between migration percentages (MP) found on MRI and conventional radiographs. Second, we analyzed the reliability of MP in MRI and radiographs.

Patients and methods — We retrospectively identified 16 patients (mean age 5 years [2–8], 14 girls), examined for RAD during a period of 2½ years. 4 raters performed blinded repeated measurements of osseous migration percentage (MP) and cartilaginous migration percentage (CMP) in MRI and radiographs. Pelvic rotation and tilt indices were measured in radiographs. Bland–Altman (B–A) plots and intraclass correlation coefficients (ICC) were calculated for agreement and reliability.

Results — B–A plots for MP_R_ and MP_MRI_ produced a mean difference of 6.4 with limits of agreement –11 to 24, with higher disagreements at low average MP values. Mean MP_R_ differed from mean MP_MRI_ (17% versus 23%, p < 0.001). MP_R_ had the best interrater reliability with an ICC of 0.92 (0.86–0.96), compared with MP_MRI_ and CMP with ICC values of 0.61 (0.45–0.70) and 0.52 (0.26–0.69), respectively. Intrarater reliability for MP_R_, MP_MRI_ and CMP all had ICC values above 0.75 and did not differ statistically significantly. Differences inMP_MRI_ and MP_R_ showed no correlation to pelvic rotation index, pelvic tilt index, or interval between radiograph and MRI exams.

Interpretation — Pelvic radiographs underestimated MP when compared with pelvic MRI. We propose CMP as a new imaging measurement, and conclude that it has good intrarater reliability but moderate interrater reliability. Measurement of MP in radiographs and MRI had mediocre to excellent reliability.

MRI scans have been used in the diagnosis and prognostication of developmental dysplasia of the hip (DDH) for the last 30 years (Bos et al. 1988). MRI scans permit the examiner complete control over orientation of the examined pelvis, allowing for more accurate measurements and visualization of non-bony structures. However, conventional pelvic radiographs are still the preferred method of examination for children over the age of 6 months due to financial considerations and challenges in scanning children, such as anxiety of the child and the need for sedation.

Residual acetabular dysplasia (RAD) occurs in 3.5–17% of treated cases of DDH (Tucci et al. 1991, Alexiev et al. 2006) and is a known risk factor for secondary osteoarthritis (Malvitz and Weinstein 1994). Indications for corrective surgery for this condition remain controversial, as seen in the tendency of surgeons to undertreat RAD patients who need surgery, rather than overtreat those who do not (Ömeroǧlu et al. 2012).

Many radiographic measurements have been proposed to indicate the severity and prognosis of DDH and many are used when deciding which patients should receive corrective surgery. The most commonly used are the osseous acetabular index (OAI) and acetabular head coverage (Ömeroǧlu et al. 2012). None of these measurements, on their own or in combination, have been shown to predict DDH prognosis accurately in all cases, and are therefore most commonly used in various combinations at the discretion of the surgeon. Acetabular head coverage, both cartilaginous and osseous, is of importance for the stability of the hip joint (Bos et al. 1991, Domenech et al. 2001) and is commonly estimated by the osseous migration percentage (MP), first proposed by Reimers (Reimers 1980), and has been shown to be predictive of later osteoarthritis (Terjesen 2011). MP estimates the percentage of the osseous femoral head that is covered by the osseous acetabulum. In this study we emulated the method developed by Reimers, in measuring the percentage of the cartilaginous femoral head covered by the cartilaginous acetabulum, and propose the name: cartilaginous migration percentage (CMP).

Currently, there are no studies comparing reliability, agreement, and correlation of MP in radiographs and MRI or between MP and CMP.

We compared agreement and correlation of MP measurements in pelvic radiographs and coronal MRI sequences. We assessed values and inter- and intrarater reliability when measuring CMP on pelvic MRI, and evaluated inter- and intrarater reliability when measuring MP on pelvic radiographs and MRI in hips evaluated for RAD corrective surgery.

## Patients and methods

This was a retrospective cohort study based on pelvic MRI and radiographs of a consecutive series of children examined for RAD at the Departments of Orthopedics and Radiology, Aarhus University Hospital (AUH), Aarhus, Denmark, during a 2½-year period from September 2016 to April 2019. Reporting follows STROBE and GRRAS statements. We included all children examined for RAD who had pelvic MRI scans done as a supplement to their previous pelvic radiographs. Exclusion criteria were: unacceptable MRI (T1 sequence not obtained, movement artifacts, relevant structures for performing each measurement not visualized) or unacceptable radiograph (relevant structures for performing each measurement not visualized due to gonadal shielding).

The examiner group consisted of 2 senior pediatric orthopedic surgeons (OR and MG) and 2 senior musculoskeletal radiologists (MBH and MH). Each examiner had at least 7 years of experience in interpreting pediatric hip radiographs. MBH had 10 years of pediatric musculoskeletal MRI experience, OR and MG each had 5 years of experience and, with only a few months’ worth, MH had the least pediatric MRI experience.

We performed measurements on T1-weighted pelvic MRI scans and the pelvic radiographs that led to the MRI scans. All MRI scans were performed at AUH where 5 different scanners were used with similar settings (Philips Medical Systems, Best, Netherlands: Achieva dStream 3.0T, Ingenia 1.5T. GE Medical Systems, Milwaukee, USA: Optima MR 450w 1.5T. Siemens Medical System, Germany: Skyra 3T, Avanto fit 1.5T). Scans were archived and viewed using Picture Archiving and Communication Software (PACS) at AUH (Impax, client 6.5 AGFA Healthcare N.V., Mortsel, Belgium). Coronal T1-weighted spin echo images were acquired. To determine the imaging sections, a transverse scout view of the acetabular region and symmetrical coronal sections was obtained. The slice thickness varied between 3 and 4 mm and the most central section was chosen. All scans were performed with the parents present and without sedation of the child. The child was placed in a supine position and had a body array coil placed anteriorly and posteriorly to the hip joint. Measurements performed on the MRI scans and AP pelvic radiographs reported in this study were: Hilgenreiner’s line, Perkins’ line, MP and CMP (Hilgenreiner 1925, Perkins 1928, Reimers 1980).

**Figure 1.**
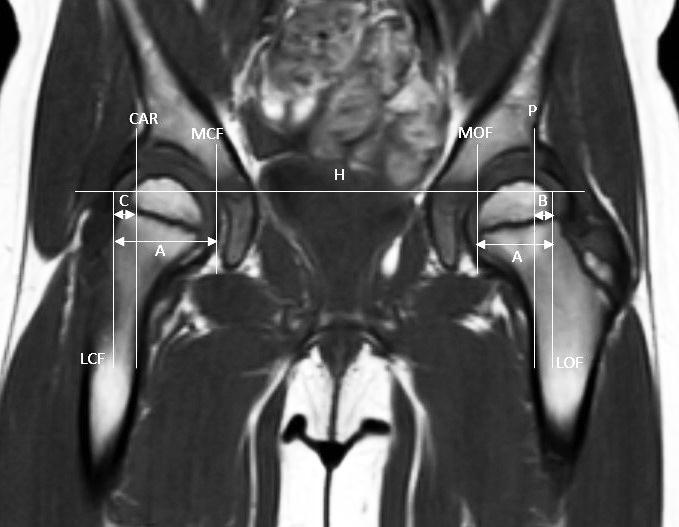
Coronal T1-weighted magnetic resonance imaging scan of pediatric hip included in this study. LOF: lateral edge of osseous femoral head, MOF: medial edge of osseous femoral head, LCF: lateral edge of cartilaginous femoral head, MCF: medial edge of cartilaginous femoral head, H: Hilgenreiner’s line, P: Perkin’s line, and CAR: vertical line through lateral edge of cartilaginous acetabular roof perpendicular to Hilgenreiner’s line. MP_MRI_ = B/AЧ100%, CMP = C/AЧ100%

To calculate CMP we measured the distance between the medial and lateral sides of the cartilaginous edge of the femoral head (A) as well as the distance between a vertical line through the most lateral aspect of the cartilaginous acetabular roof perpendicular to Hilgenreiner’s line and the lateral side of the cartilaginous edge of the femoral head (C). CMP was then calculated as: CMP = C/A x 100% (Figure 1).

A pre-study workshop was held to reach consensus on how measurements should be performed. 1 MRI scan was used for the workshop and was also included in the study. Measurements for the study began in the following weeks at the discretion of each rater. MRI sequences and image selection were noted; all data was stored in an encrypted standardized data sheet by each investigator. After a minimum of 1 week after initial rating, measurements were repeated in the same order by each investigator and stored in a secondary encrypted data sheet. Measurements were performed once before saving and raters were instructed not to edit saved measurements.

Pelvic tilt index and pelvic rotation index were based on measurements on pelvic radiographs made by 1 senior musculoskeletal radiologist (MBH) and carried out according to the specifications of Ball and Kommenda (1968) and Tönnis (1976).

Each investigator was blinded to other investigators’ measurements, their own previous measurements and all information on the patient except pelvic radiographs and MRI scans to minimize information bias. All measurements and ratings were performed independently; raters were instructed not to communicate results in any way and were aware that they would be compared with each other.

### Statistics

When evaluating the mean for each of the 3 measurement methods, both right and left hip were included for all children. Variation across individual and measured side were accounted for using a linear nested mixed model with patient ID and side as random effect, and measurement method as fixed effect, thus assuming the 4 raters were independent according to the study design. Model control was performed by investigation of the qq-plot of the model residuals.

Considerations for bilaterality were made in the ICC calculations by using 1,000 bootstrap samples for both interrater and intrarater ICC. Due to missing observations each bootstrap sample representing the total population varied in size from 13 to 16 patients. All ICC calculations were made using a 2-way, mixed-effect, single-rater ICC with absolute agreement and presented with the bootstrap mean and crude 95% confidence interval (CI). Interrater ICC was calculated using the first round of measurements with each bootstrap sample containing either all-right or all-left measurements for a specific patient. Intrarater ICC was calculated across rounds using a fixed randomly chosen rater in each bootstrap sample and a randomly chosen side for each patient.

ICC values were interpreted according to general guidelines where a value of 0 or less represents no reliability, 0.75 represents good reliability, and 1 represents complete reliability (Portney and Watkins 2012).

Spearman’s correlation coefficient was calculated among the absolute differences between radiographs and MRI and pelvic tilt, pelvic rotation, and interval. Scatter plots were investigated for any systematic association not indicated by the p-values. The plots were constructed using the absolute difference of both left and right side for all individuals measured by 1 rater (MBH) in 1 round of rating.

A B–A plot for agreement between MP_R_ and MP_MRI_ was constructed under the assumption that the differences were independent, as measurements were made and subtracted within each patient.

No sample size calculation was made; the number of patients considered for surgery for RAD at AUH during the study period determined the sample size. Statistical analyses were performed using Stata version 16.1 (StataCorp, College Station, TX, USA).

### Ethics, funding, and potential conflicts of interest

Ethical approval was not required in accordance with the guidelines of the Danish National committee on health research ethics for non-interventional studies. No external funding was obtained for this study. No conflict of interest was declared.

## Results

16 children (14 girls) were identified. 1 had missing pelvic radiographs and 1 child had incomplete MRI data, totaling pelvic radiographs and MRI scans of 30 hips. 8 hips were scanned in 3.0T scanners and 7 were scanned in 1.5T scanners, mean age at radiographic examination was 5 years (2–8), and mean interval between radiograph and MRI was 133 days (16–234). The ethnicity of included patients was: Caucasian (n = 14), Turkish (n = 1), and African (n = 1). 4 patients had musculoskeletal disorders which were: bilateral coxae vara, Calvé–Legg–Perthes disease with secondary acetabular dysplastic changes, and unilateral proximal femoral focal deficiency (Table 1).

**Table 1.** Demographics of included patients

Factor	n	Mean (SD) [range]
Time between radiographic		
and MRI examination	14	133 (72) [16–234]
Age at radiography (years)	15	5.3 (1.6) [2.1–7.9]
Age at MRI (years)	15	5.6 (1.5) [2.6–8.2]
Radiographic pelvic tilt and rotation indices		
Pelvic tilt index	15	0.73 (0.17) [0.47–1.03]
Pelvic rotation index	14	1.0 (0.14) [0.72–1.25]
Female sex	14/16	
MRI scanner		
Philips: Achieva dStream 3T	4	
Philips: Ingenia 1.5T	2	
GE: Optima MR450w 1.5T	3	
Siemens: Skyra 3T	4	
Siemens: Avanto Fit 1.5T	2	
Previous treatment for DDH		
Hip brace (Denis Browne)	2	
Closed reduction and hip spica	4	
None	8	
Unknown	2	

Mean values (CI) for the first round of rating across all raters were: MP_R_ 17 (14–20), MP_MRI_ 23 (20–26), and CMP 19 (16–22). The mean value of CMP did not differ statistically significantly from the mean of MP_R_ and MP_MRI_ (Table 2).

**Table 2.** Mean values (%) of bilateral migration measurements made in 1 round by all raters

Migration percentage	Mean (95% CI)
Osseous, radiographic (MP_R_)	17 (14–20)
Osseous, MRI (MP_MRI_)	23 (20–26)
Cartilaginous, MRI (CMP)	19 (16–22)

The mean difference between MP_MRI_ and MP_R_ was 6.4 (–15 to 38). The mean absolute difference between MP_MRI_ and MP_R_ showed no statistically significant correlation to pelvic rotation index, pelvic tilt index, or interval between examinations (Spearman’s rho 0.29, 0.35, and 0.44 respectively). Scatter plots revealed no systematic correlation.

**Figure 2.**
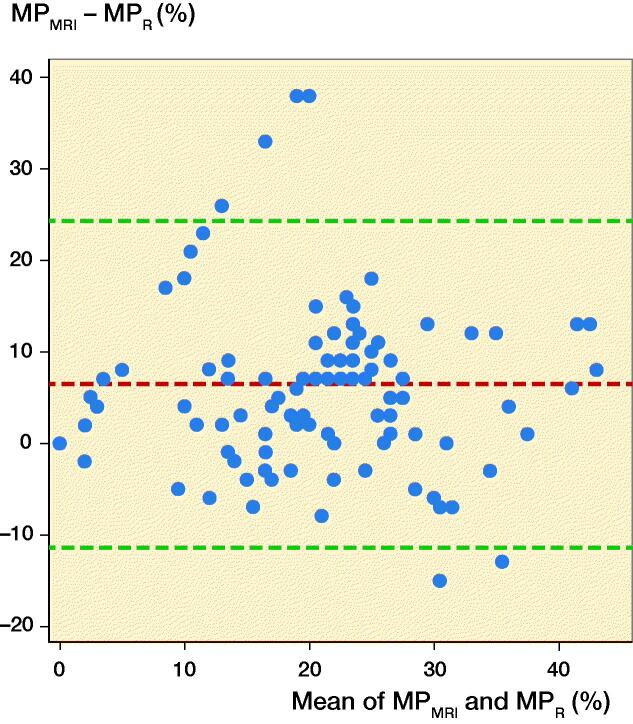
Bland–Altman plot, agreement of MP_R_ and MP_MRI_ measurements made by all raters in 1 round. Mean difference 6.4, limits of agreement –11 to 24. MP = osseous migration percentage.

The B–A plot for MP_R_ and MP_MRI_ produced a mean difference of 6.4 with limits of agreement –11 to 24, with higher disagreements at low average MP values (Figure 2).

The ICC value for MP_R_ interrater reliability was statistically significantly higher at 0.92 (0.86–0.96) when compared with MP_MRI_ and CMP, with ICC values of 0.61 (0.45–0.70) and 0.52 (0.26–0.69) respectively. No statistically significant difference was found in intrarater reliability ICC for MP_R_, MP_MRI_, and CMP with ICC values of 0.94, 0.79, and 0.82 respectively (Table 3).

**Table 3.** ICC values for inter- and intrarater reliability with 95% bootstrap confidence intervals, bootstrap samples 1,000

Migration percentage	ICC (95% CI)
Interrater reliability	
Osseous, radiographic (MP_R_)	0.92 (0.86–0.96)
Osseous, MRI (MP_MRI_)	0.61 (0.47–0.70)
Cartilaginous, MRI (CMP)	0.52 (0.26–0.69)
Intrarater reliability	
Osseous, radiographic (MP_R_)	0.94 (0.81–0.99)
Osseous, MRI (MP_MRI_)	0.79 (0.50–0.93)
Cartilaginous, MRI (CMP)	0.82 (0.41–0.97)

## Discussion

In this first study, comparing agreement, reliability, and correlation of MP in radiographs and MRI in 16 children examined for RAD, we found disagreement in MP between MRI and radiograph modalities at low values, interestingly with no correlation to pelvic orientation or interval between radiographs and MRI. Intrarater reliability in radiographs and MRI modalities across 4 independent raters was excellent and interrater reliability for the novel CMP measurement was comparable to MP_MRI_, but inferior to radiographic MP.

### Limitations

This study had some limitations. To limit selection bias, we consecutively included all patients considered for surgical intervention, at the same institution, during a period of 2½ years. However, our sample size for this rare condition was small, which translates to wide confidence intervals in our calculated means for CMP.

Acceptable ranges for pelvic tilt index and pelvic rotation index have been reported to be between 0.9–1.4 and 0.7–1.5 respectively (Yang et al. 2020). These values were exceeded for pelvic tilt index in over 80% of observations included in this study, whereas values of pelvic rotation where within acceptable limits. This reflects the clinical reality, but it has been shown to affect the accuracy of measurements on pelvic radiographs (Hamano et al. 2019), and in extension it could affect the agreement between MP_R_ and MP_MRI_. However, we found no evidence for a correlation deviating from 0 between MP_R_–MP_MRI_ and pelvic rotation or pelvic tilt.

We used 5 different MRI scanners in this study. Using higher strength scanners in musculoskeletal imaging reportedly improves image quality due to higher signal-to-noise ratios, which in turn could affect the correlation analysis of this study. However, large reviews have found insufficient evidence to link MRI scanners’ technical specifications to clinically meaningful outcomes (Wood et al. 2011). Pelvic radiographs and MRI scans were performed with a mean interval of 133 days (16–234). During this time the morphology of the examined hip could potentially have changed but we found no statistically significant correlation between the absolute mean difference, MP_R_–MP_MRI_, and the interval between radiographs and scans.

Prior to measurements, each rater participated in a workshop aimed at reaching a consensus in measurements. This was necessary as CMP is a novel measurement but reduces the independence of raters and could affect the interrater reliability and external validity of the study results.

### Interpretation

In 41 out of 235 observations, MP_R_ was measured at values of 0% and 1%, whereas the corresponding MP_MRI_ values ranged across the entire span of measurements (0–39%). This could indicate a lack of information when evaluating the pediatric hip on pelvic radiographs and shows a trend toward underestimation of MP compared with MRI.

The high level of intrarater reliability and low level of interrater reliability found in MRI measurements in this study underlines the need for proper standardized measurement guidelines.

It has long been known that the cartilaginous structures of the hip play an important part in the stability of the pediatric hip joint in DDH (Bos et al. 1988), and attempts have been made to quantify the cartilaginous acetabular head coverage. Using cartilage-optimized computed tomography (CT) scans, Lin et al. (1997) found a mean difference in coverage percentages of 17%, but this method relied on fitting anatomical structures to best-fit computer-generated geometric shapes, as CT is not optimized for visualizing cartilaginous structures. Domenech et al. (2001) proposed the acetabular head index (AHI) defined as the percentage ratio of the width of the cartilaginous femoral head covered by the cartilaginous acetabulum over the total width of the head, measured in both sagittal and coronal planes on MRI scans. The use of this parameter is not widespread, and no studies have been published on the reliability or agreement of this measurement.

Utilizing MRI, we emulated the commonly used measurement of MP developed by Reimers (1980), which is a proven prognosticator for the long-term risk for osteoarthritis (Terjesen 2011). CMP derives its measurements from the cartilaginous edge of the roof of the acetabulum, but how this measurement relates to the stability of the hip and the prognosis of DDH is currently unknown. Other measurements based on the cartilaginous edge of the acetabulum have been proposed, most notably the cartilaginous acetabular index (CAI), which is increased in DDH (Li et al. 2012) and has been proposed as a guiding measurement in the selection of patients for surgical treatment in borderline RAD cases (Merckaert et al. 2019). This could mean a future role for CAI as predictor of the developmental potential of the hip and may show promise for measurements sharing the same anatomical landmarks such as the CMP.

### Generalizability

Raters were aware that their measurements would be compared with those of their fellow raters. This awareness is subject to the Hawthorne effect, the change in subjects’ behavior due to their awareness of being observed (Chen et al. 2015), which could affect the external validity of the measurements.

### Conclusion

We compared MP measurements on MRI and pelvic radiographs and found significant disagreement, especially at low MP values. Intrarater reliability for MP and CMP across radiograph and MRI modalities was good to excellent, while interrater reliability for MRI measurements was poor. We have established the CMP as a novel MRI measurement for assessment of the pediatric hip with reliability comparable to existing MRI measurement techniques, although still inferior to the reliability of pelvic radiographs.

Future investigations into the role of CMP as a marker for the prognosis of RAD, and standardized guidelines for performing this measurement, are needed before it can be utilized in a clinical setting.
